# Microarray Analysis of Perinatal-Estrogen-Induced Changes in Gene Expression Related to Brain Sexual Differentiation in Mice

**DOI:** 10.1371/journal.pone.0079437

**Published:** 2013-11-04

**Authors:** Mototsugu Sakakibara, Yoshihisa Uenoyama, Shiori Minabe, Youki Watanabe, Chikaya Deura, Sho Nakamura, Genki Suzuki, Kei-ichiro Maeda, Hiroko Tsukamura

**Affiliations:** 1 Graduate School of Bioagricultural Sciences, Nagoya University, Nagoya, Japan; 2 Advanced Medical Science Research Department, Mitsubishi Chemical Medience Corporation, Ibaraki, Japan; 3 Molecular Genetic Research and Analysis Department, Mitsubishi Chemical Medience Corporation, Tokyo, Japan; 4 Department of Veterinary Medical Sciences, Graduate School of Agricultural and Life Science, the University of Tokyo, Tokyo, Japan; Hosptial Infantil Universitario Niño Jesús, CIBEROBN, Spain

## Abstract

Sexual dimorphism of the behaviors or physiological functions in mammals is mainly due to the sex difference of the brain. A number of studies have suggested that the brain is masculinized or defeminized by estradiol converted from testicular androgens in perinatal period in rodents. However, the mechanisms of estrogen action resulting in masculinization/defeminization of the brain have not been clarified yet. The large-scale analysis with microarray in the present study is an attempt to obtain the candidate gene(s) mediating the perinatal estrogen effect causing the brain sexual differentiation. Female mice were injected with estradiol benzoate (EB) or vehicle on the day of birth, and the hypothalamus was collected at either 1, 3, 6, 12, or 24 h after the EB injection. More than one hundred genes down-regulated by the EB treatment in a biphasic manner peaked at 3 h and 12-24 h after the EB treatment, while forty to seventy genes were constantly up-regulated after it. Twelve genes, including *Ptgds, Hcrt*, *Tmed2*, *Klc1*, and *Nedd4*, whose mRNA expressions were down-regulated by the neonatal EB treatment, were chosen for further examination by semiquantitative RT-PCR in the hypothalamus of perinatal intact male and female mice. We selected the genes based on the known profiles of their potential roles in brain development. mRNA expression levels of *Ptgds, Hcrt*, *Tmed2*, and *Nedd4* were significantly lower in male mice than females at the day of birth, suggesting that the genes are down-regulated by estrogen converted from testicular androgen in perinatal male mice. Some genes, such as *Ptgds* encoding prostaglandin D2 production enzyme and *Hcrt* encording orexin, have been reported to have a role in neuroprotection. Thus, *Ptgds* and *Hcrt* could be possible candidate genes, which may mediate the effect of perinatal estrogen responsible for brain sexual differentiation.

## Introduction

Sexual dimorphism of the behaviors or physiological functions in mammals is mainly due to the sex difference of the brain. Sex-specific patterns of endocrine function, sexual behavior and morphology have been reported in the rats. For example, female rats show cyclic release of luteinizing hormone (LH), that is a surge-mode secretion (LH surge), while males do not show the surge [1]. As sexual behaviors, male rodents show mounting behavior, while females show lordosis behavior [1]. It has been well known that the size of some nuclei in the brain show sex difference, and these nuclei are called sexual dimorphic nuclei [2]. For example, the anteroventral periventricular nucleus (AVPV) is larger in females than that in male rats [3] and the medial-anterior bed nucleus of the stria terminalis contains more neurons in female rats than do in males [4]; while the sexual dimorphic nucleus of the preoptic area (POA), accessory olfactory bulb, medial amygdala, and ventromedial hypothalamus (VMH) are larger in males than in female rats [2,4]. However, the mechanisms of brain sexual differentiation responsible for these sex differences have not been fully clarified yet.

Previous studies on sexual behavior in the rat have shown that the female-type default brain is masculinized or defeminized by estradiol converted from testicular androgens in the perinatal period in males. Female rats treated with exogenous androgen or estrogen in the neonatal period do not show lordosis in adulthood [5-11]. Male rats castrated at neonatal period show the lordosis when they are treated with estrogen at adulthood [12,13]. On the other hand, male rats neonatally treated with aromatase inhibitor, which interrupts the androgen conversion to estrogen, show lordosis behavior [14,15], whereas female rats treated with estrogen and some estrogenic compounds in the neonatal period do not [8,16]. These studies suggested that the mechanism regulating lordosis is inactivated in their neonatal period by estrogen converted from testicular androgens, resulting in loss of lordosis behavior in adult male rats. These findings suggest that male rats are equipped with the brain mechanism controlling lordosis, but the mechanism defeminized by neonatal estrogen converted from testicular androgen.

Previous studies have suggested that the kisspeptin neurons located in the AVPV of the anterior hypothalamus regulate gonadotropin-releasing hormone (GnRH)/LH surge [17-21], and that defeminization of the mechanism governing the GnRH/LH surge system is mainly due to the defeminization of the AVPV kisspeptin neurons in the rodent [21-24]. The mechanisms of estrogen action resulting in the defeminization of the kisspeptin neurons have not been clarified yet. Thus, large-scale analysis by microarray would be a powerful tool to explore the candidate genes mediating the effect of estrogen responsible for the brain sexual differentiation.

The present study aimed to clarify the mechanism of sexual differentiation of the brain. We analyzed the time course of the estrogen-responsive gene expression profiles in the neonatal female mouse hypothalamus to determine target genes of short-term and long-term estrogen organizational effects responsible for sexual differentiation of the brain. Microarray analysis was conducted for exhaustive identification of the genes mediating the perinatal estrogen action. We focused on several candidate genes with special attention to the gene function, such as neuronal death, neuroprotection, and development. The difference in the expression of candidate genes obtained by the analysis was examined between male and female mice at the perinatal period . 

## Materials and Methods

### Animals

Female neonatal C57BL/6J (B6) mice were used for microarray analysis to obtain candidate genes related to sexual differentiation of the brain induced by estrogen. Male and female perinatal Crlj:CD1 (ICR) mice were used for semiquantitative reverse transcriptase-polymerase chain reaction (RT-PCR) analysis to determine the sex differences in expressions of the candidate genes obtained by the microarray analysis. Animals were housed under a 14 : 10 h light/dark cycle (lights on 05.00 h) at 23°C ± 2°C and were provided with standard rodent chow (OA-2 for B6; CE-2 for ICR, CLEA, Tokyo, Japan) and water *ad lib*. The day of parturition and 2 days after the birth was designated day 0 (D0) and day 2 (D2), and the embryonic day 16 and 18 were designated E16 and E18, respectively. The present study was approved by the Committee on Animal Experiments of the Graduate School of Bioagricultural Sciences, Nagoya University.

### Microarray Analysis of Neonatal Estrogen Effect on Gene Expressions in the Hypothalamus

B6 female mice (approximately 1.4 g BW, n=3 in each group) were subcutaneouly injected with either estradiol benzoate (EB, 7.5 μg/20 μl peanut oil, that is equivalent to 5.4 μg/g BW) or vehicle on the D0, and the whole hypothalamus was collected at either 1, 3, 6, 12 or 24 h after the EB injection to explore time-dependent changes in estrogen-induced gene expressions in the hypothalamus. The dose was chosen according to the previous studies which showed that single injection of EB (2.5-12.5 μg/g BW) in neonatal female rats defeminized the sexual behavior [7-9], GnRH/LH surge mechanism [25] , and the AVPV *Kiss1* expression [24]. Therefore, the dose of 7.5 μg of EB for D0 in the present study was thought to be enough for defeminization of the GnRH/LH surge-regulating mechanism. Animals were sacrificed by decapitation, and then the hypothalamus was collected by dissecting anterior to the optic chiasm and caudally at the mammillary bodies under the microscope. Total RNA of the whole hypothalamus containing some nuclei, such as the AVPV, POA, VMH and arcuate nucleus, was isolated with RNeasy mini kit according to the manufacturer’s instructions (QIAGEN, Hilden, Germany). RNAs derived from the hypothalamus of individual were reversetranscribed, labeled with biotin, and analyzed using the GeneChip Mouse Genome 430 2.0 array (Affymetrix, Santa Clara, CA). Arrays in each animal were processed according to the manufacturer’s instructions. Microarray data have been deposited in Gene Expression Omnibus (GEO) database, www.ncbi.nlm.nih.gov/geo/ (accession no. GSE47798).

GeneSpring GX 7.3 software (Agilent Technologies, Palo Alto, CA) was used to identify the difference in gene expression between EB- and vehicle-treated neonatal mice. The threshold of gene expression to select candidate genes mediating the effect of the EB treatment on the developing brain was as follows: 2-fold or greater in EB-treated mice than vehicle-treated mice (increased gene expression); 0.5-fold or less in EB-treated mice than vehicle-treated mice (decreased gene expression) at each time point. Those candidate genes were mapped to Entrez Gene IDs and clustered in Gene Ontology (GO) terms (version 1.2, http://www.geneontology.org/) using WEB-based GEne SeT AnaLysis Toolkit (http://bioinfo.vanderbilt.edu/webgestalt/) with the following criteria: reference set for enrichment analysis, mouse genome; statistical method, hypergeometric; multiple test adjustment, Benjamini & Hochberg method; signficance level, less than 0.05; number of genes for a category, equal to or more than two.

We selected 12 genes (3 genes from 3 h, 4 genes from 12 h, and 5 genes from 24 h after EB injection in experimental groups) based on the GO analysis and the known profiles of their potential roles, such as apoptosis, neuroprotection, cell-differentiation, and intra/extracellular component proteins for further investigation in the semiquantitative RT-PCR experiment.

#### Detection of Sex Difference of Gene Expressions in the Hypothalamus by Semiquantitative RT-PCR Analysis

The hypothalamus taken from male and female ICR mice on E16, E18, D0, or D2 was used for semiquantative RT-PCR analysis, according to the results of microarray analysis. Total RNA isolated from the hypothalamus (n=4 in each group) was processed using ISOGEN (Nippon Gene Co., Tokyo, Japan) according to the manufacturer’s instructions. Two μg of RNA was used to reverse transcribe using high-capacity cDNA of the reverse transcription kit RT (Life Technologies, Foster City, CA) in accordance with the instructions. All primer sequences and PCR products are described in Table S1. The following 12 genes were analyzed by semiquantative RT-PCR: transmembrane emp24 domain trafficking protein 2 (*Tmed2*), an intracellular transporter [26]; kinesin light chain 1 (Klc1), an intracellular transporter [27]; neural precursor cell-expressed developmentally down-regulated 4 (*Nedd4*), an ubiquitin ligases [28]; hypocretin (*Hcrt*), an orexigenic peptide [29]; Meis homeobox 2 (*Meis2*), a transcription factor [30]; neurogenic differentiation 6 (*Neurod6*), a neurogenic transcription factor [31]; paired-like homeodomain transcription factor 2 (*Pitx2*), a transcription factor related to neuron differentiation [32]; apolipoprotein D (*Apod*), a local transporter of steroid hormones in the brain [33]; vitronectin (*Vtn*), a glycoprotein supporting axonal growth [34]; prostaglandin D2 (PGD2) synthase (*Ptgds*), a brain-type PGD2 synthase [35]; fibronectin 1 (Fn1), an extracellular matrix protein [36]; insulin-like growth factor 2 (Igf2), a growth factor [37]. Gene expressions of estrogen receptor α (*Est1*) and progesteron receptor (Pgr) were also analyzed as positive controls, because those gene expressions were affected by perinatal steroids [38]. *Est1* and *Pgr* expressions were lower and higher in postnatal male mice than females, respectively, as previously reported (Figure S1). β-Actin (*Actb*) was used as an internal control. RT-PCR for the mRNAs for *Actb* was performed under the following conditions: 95°C for 5 min, and followed by 35 cycles at 94°C for 30 sec, 60°C for 1 min, and 72°C for 1 min using AmpliTaq Gold DNA polymerase (Life Technologies). PCR for *Tmed2*, *Klc1*, *Nedd4*, *Hcrt*, *Meis2*, *Apod*, *Vtn*, *Ptgds*, Fn *1* and *Igf2* was performed under the following conditions: 95°C for 5 min, followed by 30 cycles at 94°C for 30 sec, 60°C for 1 min, and 72°C for 1 min using AmpliTaq Gold DNA polymerase. PCR for *Neurod6* and *Pitx2* was performed under the following conditions: 95°C for 5 min, followed by 30 cycles at 94°C for 30 sec, 62°C for 1 min, and 72°C for 1 min using AmpliTaq Gold DNA polymerase. PCR for *Est1* and *Pgr* was performed under the following conditions: 95°C for 2 min, followed by 30 cycles at 98°C for 20 sec, 60°C for 30 sec, and 68°C for 1 min using KOD-FX (Toyobo Co., Osaka, Japan). mRNA levels of *Tmed2*, *Klc1*, *Nedd4*, *Hcrt*, *Meis2*, *Neurod6, Pitx2*, *Apod*, *Vtn*, *Ptgds*, Fn *1* and *Igf2* were semiquantified using the Image J program (version 1.44o; http://imagej.nih.gov/ij). The intensity was then expressed as a value relative to that of the *Actb* amplicon. 

### Statistical Analysis

Statistical differences in signal intensity examined by the microarry between EB- and vehicle-treated female mice were determined by two-way ANOVA (treatment and time as main factors) followed by the Bonferroni test. Statistical differences in mRNA levels between male and female mice examined by the semiquantitative RT-PCR were also determined by two-way ANOVA (sex and age as main factors) followed by the Bonferroni test.

## Results

### Microarray Analysis of Neonatal Estrogen Effect on Gene Expressions in the Hypothalamus

**Figure 1:**
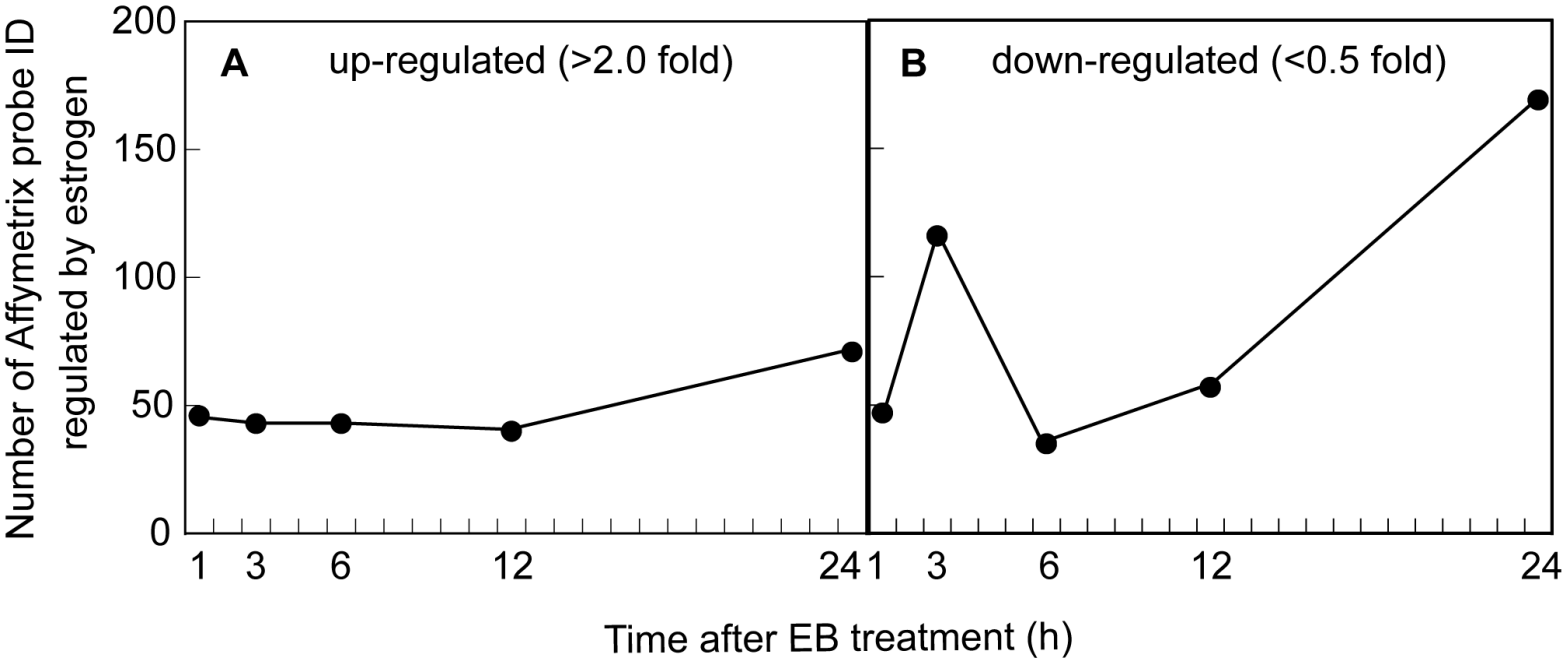
The number of Affymetrix probe ID, expressions of which were increased or decreased by neonatal estradiol bezoate (EB) treatment, in the hypothalamus of female mice. A, Numbers of genes showing a 2-fold or greater expression in EB-treated group compared with vehicle-treated control group; B, Numbers of genes showing a 0.5-fold or less expression in EB-treated group compared with vehicle-treated control group. Female mice were subcutaneously treated with EB (7.5 μg) on the day of birth and then the hypothalamus were collected at either 1, 3, 6, 12,or 24 h after the EB treatment. Difference of gene expression between EB- and vehicle-treated control mice was determined by GeneSpring GX 7.3 software.

**Figure 2:**
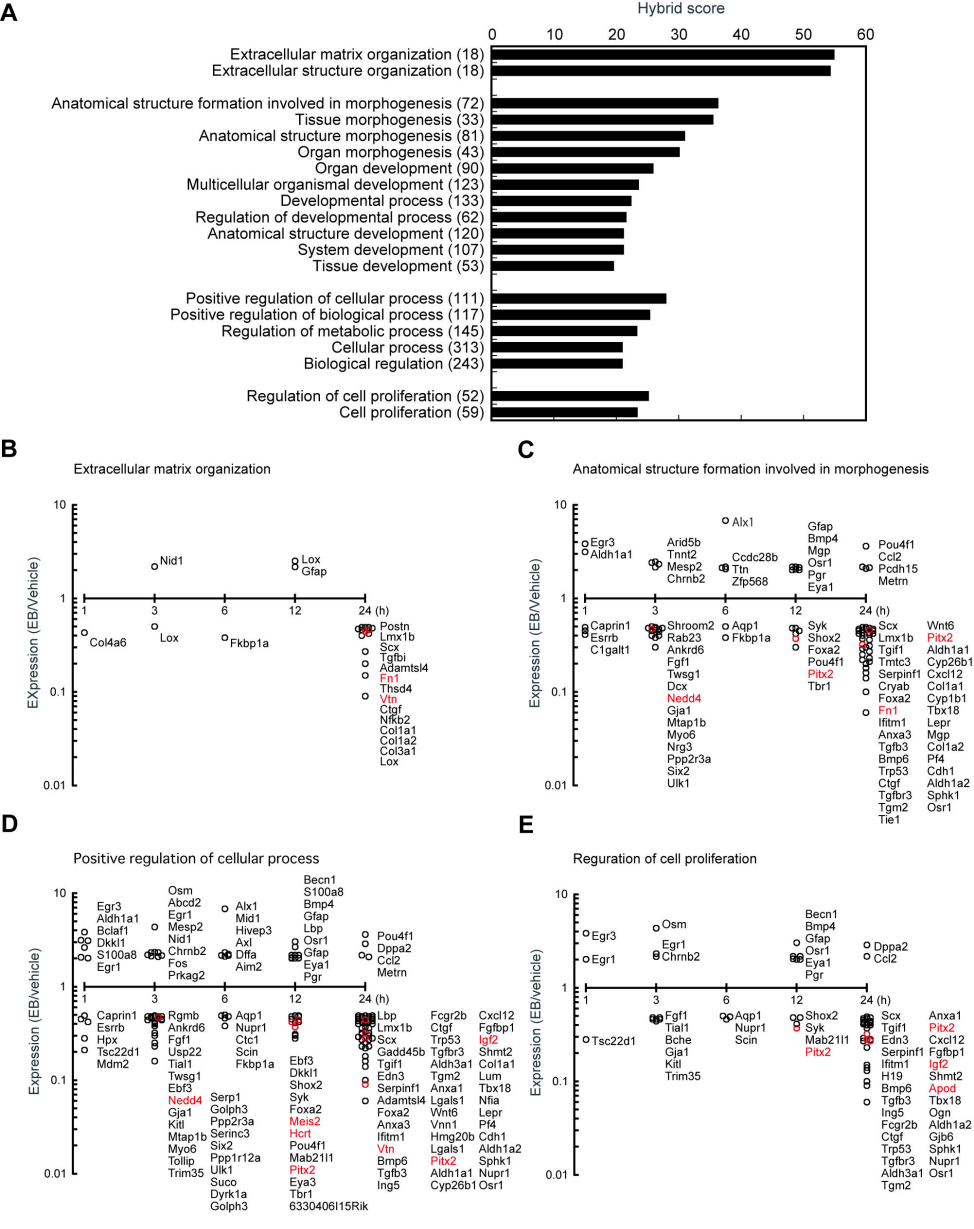
Gene ontology enrichment analysis for the genes differentially expressed between EB- and vehicle-treated neonatal mouse hypothalamus. The whole hypothalamus was collected from neonatal female mice 1, 3, 6, 12, and 24 h after EB or vehicle injection. The 478 unique Entrez Gene IDs were analyzed by a biological process gene ontology analysis. Significant regulated 20 gene ontology terms were shown in A. Time-dependent changes in gene expressions were depicted for 4 representative gene ontology terms such as the extracellular matrix organization (B), anatomical structure formation involved in morphogenesis (C), positive regulation of cellular process (D), and regulation of cell proliferation (E). Gene symbols in red were selected for further investigation to evaluate sex difference in expression levels by a semiquantitative RT-PCR.

To acquire a general overview of gene expression by neonatal EB treatment, transcripts of each time points were plotted in the form of a heatmap (Figures S2 - S6). Heatmaps showed that a number of genes were down-regulated by 3 h-and 24 h-EB treatment (Figures S2 - S6). Figure 1 shows two peaks 3 h and 24 h after EB treatment in the numbers of down-regulated genes (less than 0.5 fold-change vs vehicle-treated control, Figure 1B), whereas numbers of up-regulated genes (more than 2.0 fold-change vs vehicle-treated control, Figure 1A) were stable through each time point. Out of a total of 667 Affymetrix probe set IDs, 539 IDs were unambiguously mapped to 478 unique Entrez Gene IDs. The remaining 128 IDs were mapped to multiple Entrez Gene IDs or could not be mapped to any Entrez Gene ID. The 478 unique Entrez Gene IDs were clustered in the GO biological process terms. As shown in Figure 2A, significant regulated 20 gene ontology terms were classified into extracellular matrix/structure organization, some branches of developmental process, branches of the biological regulation and cellular process, and cell proliferation. Of those, 18 genes clustered in the extracellular matrix organization, 72 genes clustered in the anatomical structure formation involved in morphogenesis, 111 genes clustered in the positive regulation of cellular process, and 52 genes clustered in the regulation of cell proliferation were depicted in Figure 2B-E. *Pgr* expression in the EB-treated female hypothalamus was two times higher than vehicle-treated controls (Figure 2 C-E). This is consistent with the previous study [39] in which progesterone receptor expression is higher in male mice than females, but kept low in estrogen receptor α-null male mice. 

**Figure 3:**
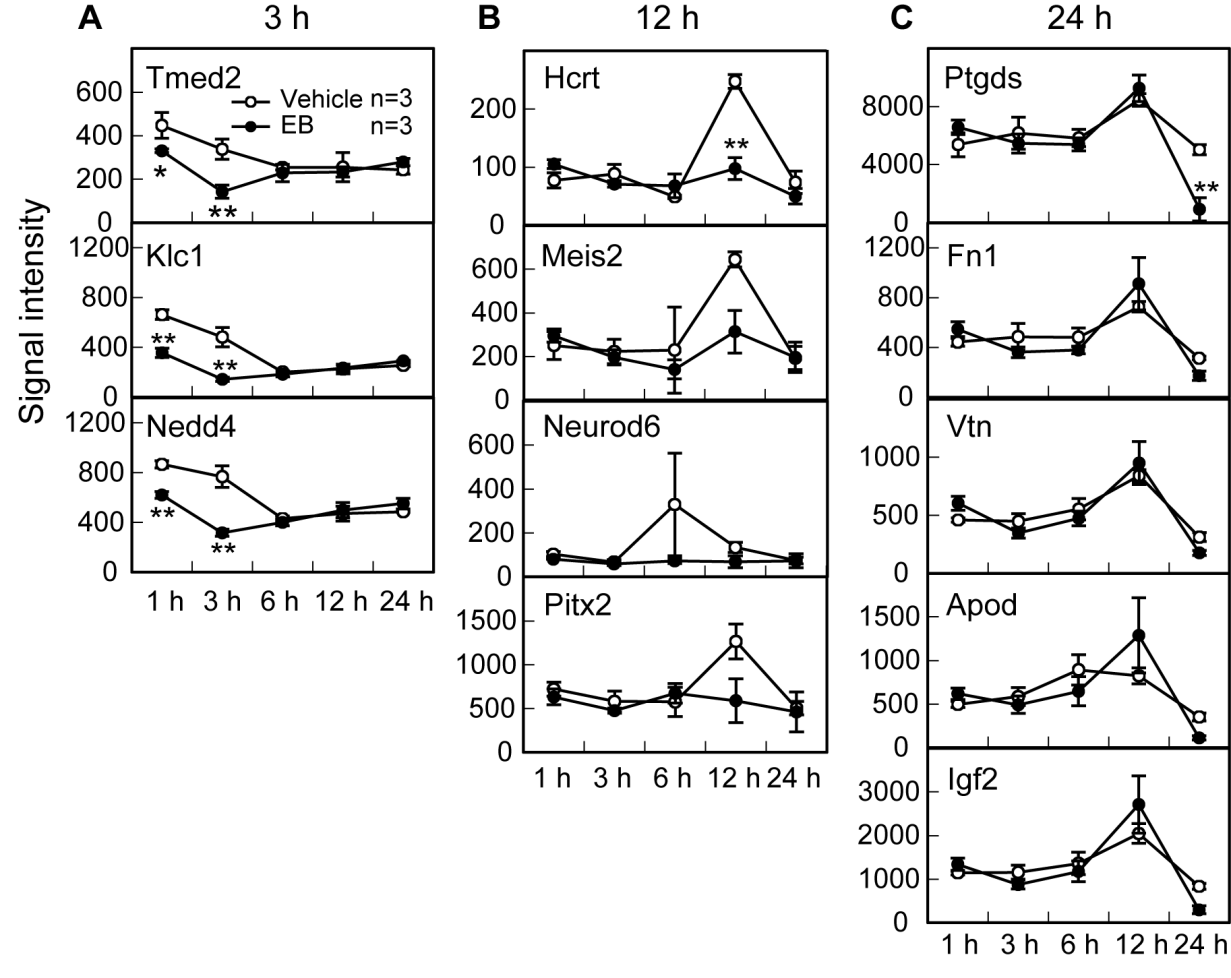
Changes in expressions of *Tmed2*, *Klc1*, *Nedd4, Hcrt*, *Meis2*, *Neurod6*, *Pitx2, Ptgds*, *Vtn*, Fn *1*, *Apod*, and *Igf2* genes in the female mouse hypothalamus 3 h (A), 12 h (B), or 24 h (C) after the EB treatment. Values in EB-treated (solid circle) and vehicle-treated controls (open circle) are indicated as signal intensity in microarray hybridization. Values are means±SEM. Values marked with asterisks (* or **) are significantly different from vehicle-treated controls at each time point (P < 0.05 or P < 0.01, two-way ANOVA (treatment and time as main factors) followed by the Bonferroni test).

Two-way ANOVA analysis (main factors, treatment and time) revealed the significant effects of time (F4,20 = 17.6, P < 0.01) and an interaction between treatment and time (F4,20 = 4.4, P < 0.05) on *Ptgds* expression level in the whole mouse hypothalamus (Figure 3). The signal intensity of *Ptgds* gene expressions after 24 h EB treatment was significantly (P < 0.05, Bonferroni) lower than that of vehicle treatment. Two-way ANOVA revealed the significant effects of treatment (F1,20 = 10.5, P < 0.05), time (F4,20 = 21.8, P < 0.01) and an interaction between treatment and time (F4,20 = 12.8, P < 0.01) on the *Hcrt* expression level (Figure 3). The *Hcrt* levels were significantly lower in EB-treated mice than in vehicle-treated mice 12 h after the injection. Two-way ANOVA revealed the significant effects of treatment (F1,20 = 7.4, P < 0.05), time (F4,20 = 5.8, P < 0.05) and an interaction between treatment and time (F4,20 = 2.9, P < 0.05) on the *Tmed2* expression level (Figure 3). The analysis revealed the significant effects of treatment (F1,20 = 31.6, P < 0.01), time (F4,20 = 25.0, P < 0.01) and an interaction between treatment and time (F4,20 = 13.8, P < 0.01) on the *Klc1* expression level (Figure 3). The analysis also revealed the significant effects of treatment (F1,20 = 20.0, P < 0.01), time (F4,20 = 15.0, P < 0.01) and an interaction (F4,20 = 11.8, P < 0.01) on the *Nedd4* expression level (Figure 3). The *Tmed2*, *Klc1* and *Nedd4* levels were significantly lower in EB-treated mice than vehicle-treated mice 1 and 3 h after the injection. Two-way ANOVA analysis revealed a significant effect of time on the expression levels of *Meis2* (F4,20 = 4.4, P < 0.05), Fn *1* (F4,20 = 12.3, P < 0.01), *Vtn* (F4,20 = 18.8, P < 0.01), *Apod* (F4,20 = 6.7, P < 0.01), and *Igf2* (F4,20 = 13.2, P < 0.01) genes in the hypothalamus of perinatal mice. The analysis revealed no significant effect of treatment and time on the expression levels of *Neurod6* and *Pitx2* genes in the hypothalamus of perinatal mice.

#### Sex Difference in Candidate Gene Expressions in Perinatal Mice

**Figure 4:**
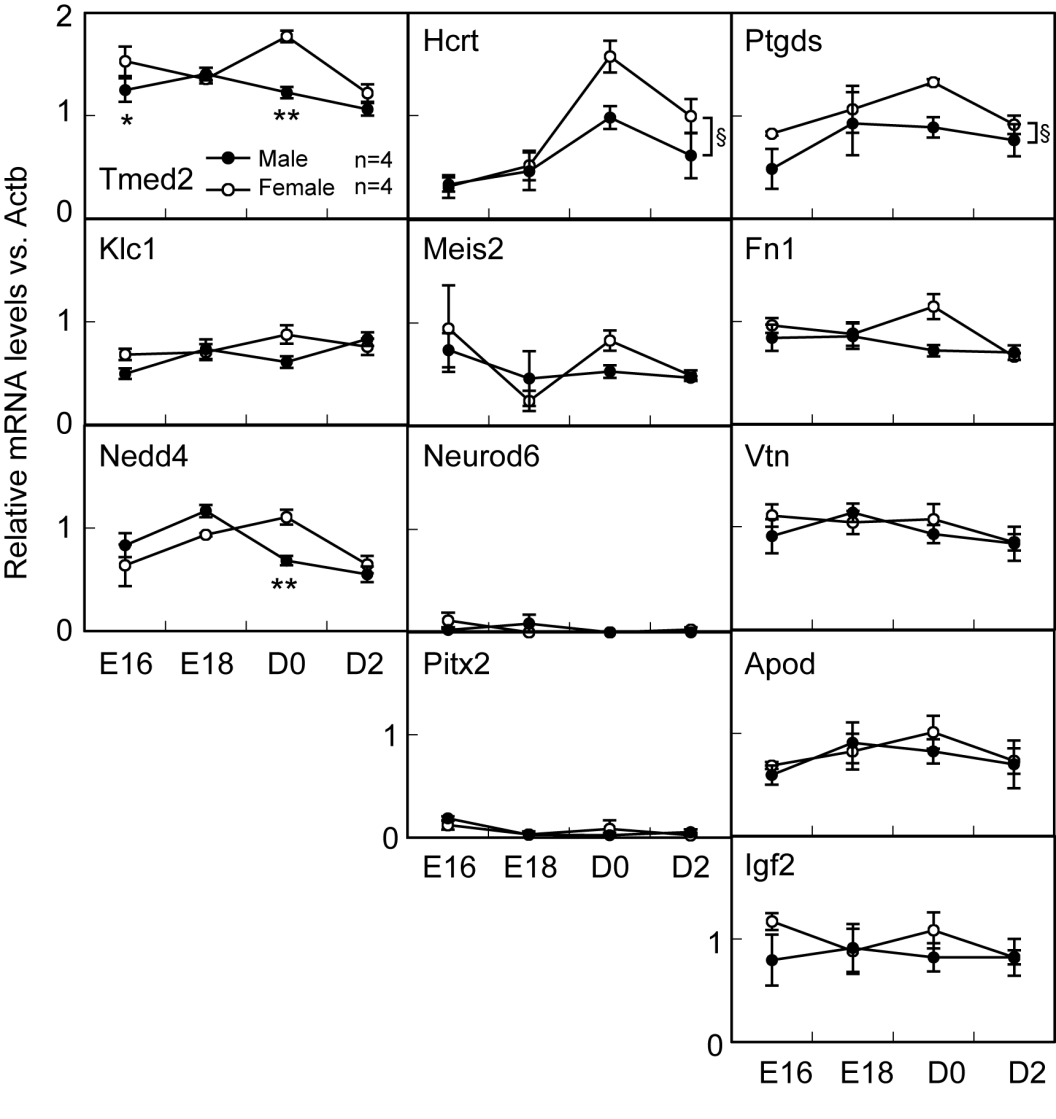
Expressions of *Tmed2*, *Klc1*, *Nedd4, Hcrt*, *Meis2*, *Neurod6*, *Pitx2, Ptgds*, *Vtn*, Fn *1*, *Apod*, and *Igf2* genes in the hypothalamus of perinatal intact male and female mouse at embryonic day 16 (E16), E18, the day of birth (D0), and two days after the birth (D2). mRNA levels of these genes were determined semiquantitatively by RT-PCR followed by analysis with Image J from NIH. Gene expression levels in intact male (solid circle) and female hypothalamus (open circle) were indicated in relation to *Actb*. Values are means±SEM. Values marked with asterisks (* or **) are significantly different from female mice at each time point (P < 0.05 or P < 0.01, two-way ANOVA (sex and age as main factors) followed by the Bonferroni test). Section signs indicate significant effect between sex as a main factor of two-way ANOVA.

Two-way ANOVA analysis (main factors, sex and age) revealed the significant effects of sex (F1,24 = 5.1, P < 0.05) on *Ptgds* expression level in the whole mouse hypothalamus (Figure 4). The analysis revealed the significant effects of sex (F1,24 = 5.6, P < 0.05), age (F3,24 = 15.5, P < 0.01) on the *Hcrt* expression level (Figure 4). The *Ptgds* and *Hcrt1* levels were significantly lower in male than female mice. The analysis revealed the significant effects of sex (F1,24 = 15.6, P < 0.01), age (F3,24 = 6.5, P < 0.01) and an interaction between sex and age (F3,24 = 4.4, P < 0.05) on the *Tmed2* expression level (Figure 4). *Tmed2* levels were significantly lower in male than female mice at E16 and D0. The analysis showed the significant effects of age (F3,24 = 7.9, P < 0.01) and an interaction (F3,24 = 4.8, P < 0.01) on the *Nedd4* expression level (Figure 4). The *Nedd4* levels were significantly lower in male than female mice at D0, whereas the levels were significantly higher in male than in female mice at E18. The analysis showed the significant effects of age (F3, 24 = 4.1, P < 0.05) on the *Pitx2* expression level. Two-way ANOVA analysis disclosed no significant effect of sex and age on the expression levels of *Klc1*, *Meis2*, *Neurod6*, Fn *1*, *Vtn*, *Apod*, and *Igf2* genes in the hypothalamus of perinatal mice. *Pgr* expression was significantly higher in the hypothalamus of D2 male mice than age-matched females, whereas *Esr1* expression was significantly higher in females than age-matched males (Figure S1).

## Discussion

In the present study a large-scale analysis was made of changes in gene expression in the brain induced by neonatal estrogen treatment in female mice. The results obtained by this approach may provide directions for future investigations into the understanding of the mechanism underlying the sexual differentiation of the brain. We found that more than one hundred genes were down-regulated by neonatal estrogen in a biphasic manner with peaks 3 h and 24 h after estrogen treatment, while forty to seventy genes were constantly up-regulated 1-24 h after estrogen treatment. We focused on down-regulated genes to identify the candidate genes related to brain sexual differentiation, and then found that expressions of some genes were lower in the neonatal male mice than females. We selected the 12 genes based on their known profiles of potential roles, such as neuronal death/neuroprotection and neuronal development (Figure 3). Among these 12, expressions of 4 genes (*Tmed2*, *Nedd4*, *Hcrt*, and *Ptgds*) in neonatal male hypothalamus were lower than those in females, suggesting that the gene expressions are down-regulated by endogenous estrogen at the neonatal stage in a physiological condition. Indeed, the 4 genes were not listed in the previous microarray study, which reported that over fifty genes showed differential expression between the brains of male and female mice at embryonic 10.5 days before any gonadal hormone influence [35]. Thus, the lower expression of the candidate genes in the male mouse hypothalamus could be due to the influence of perinatal androgen secretion.

Expression of *Ptgds*, a gene encoding brain-type PGD2 synthase, was down-regulated 24 h after estrogen treatment and lower in the perinatal male mice compared with females. The *Ptgds* located in the rostral POA close to the AVPV was down-regulated by estrogen in adult female mice [40]. Hadjimarkou et al. showed that the PGD2 synthase expression in the ventrolateral POA was down-regulated by estrogen in adult female rats [41]. They also demonstrated that there is a sex difference in the PGD2 synthase expression in adult rats and suggested that organizational effects of gonadal hormones caused this sex difference. *In vitro* study suggested that PGD2 plays a neuroprotection role in the primary cultured hippocampal cells taken from embryonic day 18 female rats [42]. The AVPV, a sexual dimorphic nucleus, has been acknowledged to be responsible for the sexual dimorphism of GnRH/LH surge system [21,24]. It is, therefore, speculated that *Ptgds* could be involved in defeminizing the sexual dimorphic nuclei such as the AVPV in mice. *Hcrt*, a gene encoding hypocretin/orexin, could be a candidate gene involved in sexual differentiation of the brain in mice, because the gene expression was down-regulated 12 h after estrogen treatment and lower in perinatal male mice compared with females. Hypocretin/orexin, an orexigenic peptide, has also been reported to play a neuroprotective role in the primary cultured cortex neuron of embryonic day 17 rats [43]. Thus, it is possible that *Hcrt* could be involved in the brain sexual differentiation of the mouse brain by its neuroprotectional effect. 

The lower expressions of *Tmed2* and *Nedd4* in D0 male mouse hypothalamus than females were consistent with the results obtained by the current microarray analysis, which shows expressions of these genes were down-regulated by neonatal estrogen treatment. The functions of these genes have been reported as follows: *Tmed2* reportedly encode an intracellular transporter protein [26]; *Nedd4* has been found to encode an ubiquitin ligase [28]. Considering the known functions of these genes, *Tmed2* and *Nedd4* may have a potential function related to the brain sexual differentiation. We found no sex difference in expressions of *Klc1*, *Meis2*, *Neurod6*, *Pitx2*, Fn *1*, *Apod*, *Vtn*, and *Igf2* in the hypothalamus of perinatal mice, although the gene expressions were lower in EB-treated female mice than those of vehicle-treated controls in microarray analysis. In this case, the EB treatment may have caused extra-physiological effects on these genes. It is unlikely that *Klc1*, *Meis2*, *Neurod6*, *Pitx2*, Fn *1*, *Apod*, *Vtn*, and *Igf2* are involved in brain sexual differentiation.

Most of the above-mentioned genes, such as *Ptgds*, *Hcrt*, *Tmed2* and *Nedd4*, showed lower expressions in male mice at D0 than females. The down-regulation of these genes at D0 in male mice could be due to estrogen converted from perinatal androgen. This notion is consistent with the previous studies showing that the serum testosterone level at 0-4 hr after the birth is higher in male mice than females [44]. McCarthy [45] previously suggested that γ-aminobutyric acid (GABA), glutamate, and prostaglandin E2 could be involved in brain sexual differentiation. However, our microarray analysis did not find that neonatal estrogen affects the expressions of *Gad* encoding glutamic acid decarboxylase: a GABA synthase, and *Ptgs2* encoding prostaglandin-endoperoxide synthase 2 (Cox-2): a prostaglandin synthase. The current study evaluated sex differences in gene expressions in the whole hypothalamus in mice, while McCarthy tested sex differences in gene and protein expressions in the discrete hypothalamic regions, such as POA, in rats. The discrepancy between their study and ours could be due to the difference in the brain region and species.

It has been suggested that sexual differentiation of the POA in the rodent is mediated by the complex interplay of diverse hormonally regulated mechanisms, including cell survival and death, neurogenesis, cell morphological differentiation, as well as epigenetic control of gene expression [46]. Epigenetic programming in the developmental period is suggested to be involved in the sex difference (higher in female than male) in estrogen receptor α expression in the rat POA [47]. Bax-mediated apoptosis is known to cause sexual differentiation in cell number of sexually dimorphic nuclei, such as the AVPV and the bed nucleus of the stria terminalis [48]. It is possible that the above-mentioned genes, such as *Ptgds*, *Hcrt*, *Tmed2* and *Nedd4*, could be involved in an epigenetic or apoptotic mechanism underlying sexual differentiation of the neonatal brain.

We found that expression of some genes related to the extracellular organization, morphogenesis, regulation of cellular process and cell proliferation were up-regulated by neonatal EB treatment in female mice (Figure 2). This indicates that the genes could be involved in the sexual differentiation of the brain. Further studies are required to address this issue.

In summary, the present study demonstrated that neonatal estrogen affected expressions of a number of genes in the developing mouse brain. Expressions of some candidate genes, such as *Ptgds* and *Hcrt* in the hypothalamus were down-regulated by neonatal estrogen treatment in female mice, which is consistent with the sex difference of these gene expressions: lower in neonatal intact male mice than females. Thus, *Ptgds* and *Hcrt* could be candidate genes, which may mediate the effect of perinatal estrogen responsible for brain sexual differentiation. Further studies are required to closely investigate the precise mechanism of the candidate genes. 

## Supporting Information

Figure S1 **Expressions of Esr1 and Pgr genes in the hypothalamus of perinatal intact male and female mouse at E16, E18, D0, and D2.** mRNA levels of these genes were determined semiquantitatively by RT-PCR followed by analysis with Image J from NIH. Gene expression levels in intact male (solid circle) and female hypothalamus (open circle) were indicated in relation to *Actb*. Values are means±SEM. Values marked with asterisks (* or **) are significantly different from those in female mice (P < 0.05 or P < 0.01), two-way ANOVA (sex and age as main factors) followed by the Bonferroni test).
(TIF)

Figure S2 **Transcriptional response to 1-h estradiol benzoate (EB) treatment in the hypothalamus of neonatal female mice.** Heat map shows the hierarchical clustering of 93 Affymetrix probe ID either increased or suppressed by 2-fold or more in the whole hypothalamus of neonatal female mice 1 h after subcutaneous EB injection compared with vehicle injection. Each column represents a individual mouse and each row represents a single Affymetrix probe ID. Red indicates increased gene expression while green indicates decreased gene expression relative to median of vehicle treated controls, as indicated in the scale bar.
(TIF)

Figure S3 **Transcriptional response to 3-h EB treatment in the hypothalamus of neonatal female mice.** Heat map shows the hierarchical clustering of 159 Affymetrix probe ID either increased or suppressed by 2-fold or more in the whole hypothalamus of neonatal female mice 3 h after subcutaneous EB injection compared with vehicle injection. See Figure S2 for details.
(TIF)

Figure S4 **Transcriptional response to 6-h EB treatment in the hypothalamus of neonatal female mice.** Heat map shows the hierarchical clustering of 78 Affymetrix probe ID either increased or suppressed by 2-fold or more in the whole hypothalamus of neonatal female mice 6 h after subcutaneous EB injection compared with vehicle injection. See Figure S2 for details.
(TIF)

Figure S5 **Transcriptional response to 12-h EB treatment in the hypothalamus of neonatal female mice.** Heat map shows the hierarchical clustering of 97 Affymetrix probe ID either increased or suppressed by 2-fold or more in the whole hypothalamus of neonatal female mice 12 h after subcutaneous EB injection compared with vehicle injection. See Figure S2 for details.
(TIF)

Figure S6 **Transcriptional response to 24-h EB treatment in the hypothalamus of neonatal female mice.** Heat map shows the hierarchical clustering of 240 Affymetrix probe ID either increased or suppressed by 2-fold or more in the whole hypothalamus of neonatal female mice 24 h after subcutaneous EB injection compared with vehicle injection. See Figure S2 for details.
(TIF)

Table S1 **Sequence of primers used for RT-PCR.**
(DOC)
