# Supplementary figures and images for: Microarray Analysis of Perinatal-Estrogen-Induced Changes in Gene Expression Related to Brain Sexual Differentiation in Mice
Source: PLoS One. 2013 Nov 4;8(11):e79437. doi: 10.1371/journal.pone.0079437 (PMC3817063; doi:10.1371/journal.pone.0079437)

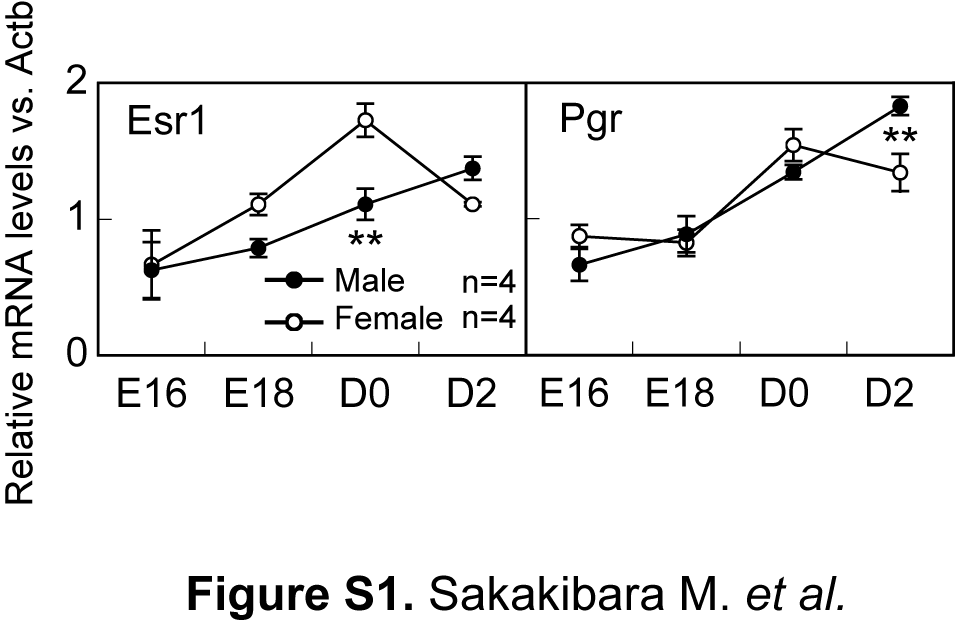

Supplement: Figure S1 — Expressions of Esr1 and Pgr genes in the hypothalamus of perinatal intact male and female mouse at E16, E18, D0, and D2. mRNA levels of these genes were determined semiquantitatively by RT-PCR followed by analysis with Image J from NIH. Gene expression levels in intact male (solid circle) and female hypothalamus (open circle) were indicated in relation to Actb. Values are means±SEM. Values marked with asterisks (* or **) are significantly different from those in female mice (P < 0.05 or P < 0.01), two-way ANOVA (sex and age as main factors) followed by the Bonferroni test). (TIF) [file pone.0079437.s001.tif]

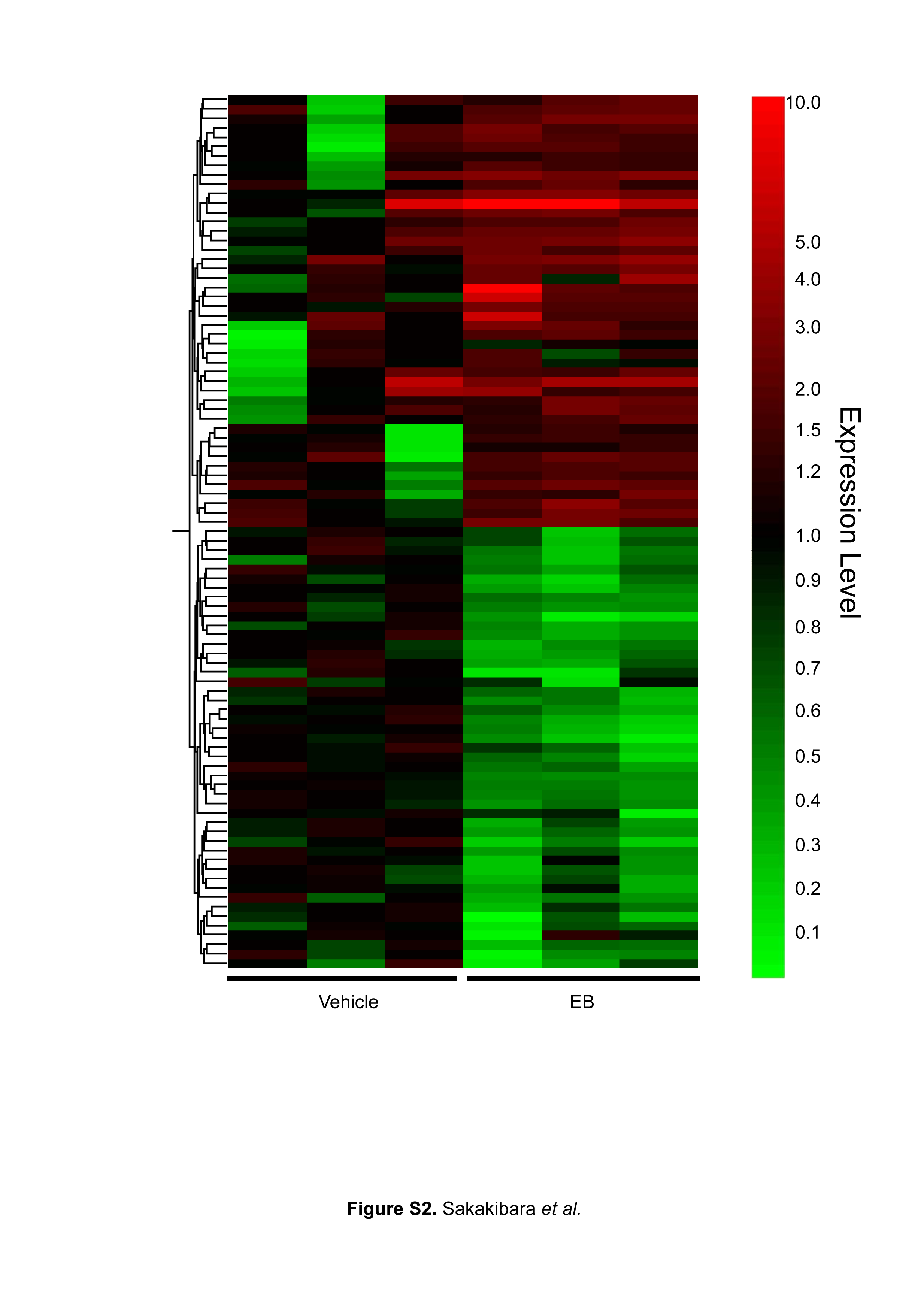

Supplement: Figure S2 — Transcriptional response to 1-h estradiol benzoate (EB) treatment in the hypothalamus of neonatal female mice. Heat map shows the hierarchical clustering of 93 Affymetrix probe ID either increased or suppressed by 2-fold or more in the whole hypothalamus of neonatal female mice 1 h after subcutaneous EB injection compared with vehicle injection. Each column represents a individual mouse and each row represents a single Affymetrix probe ID. Red indicates increased gene expression while green indicates decreased gene expression relative to median of vehicle treated controls, as indicated in the scale bar. (TIF) [file pone.0079437.s002.tif]

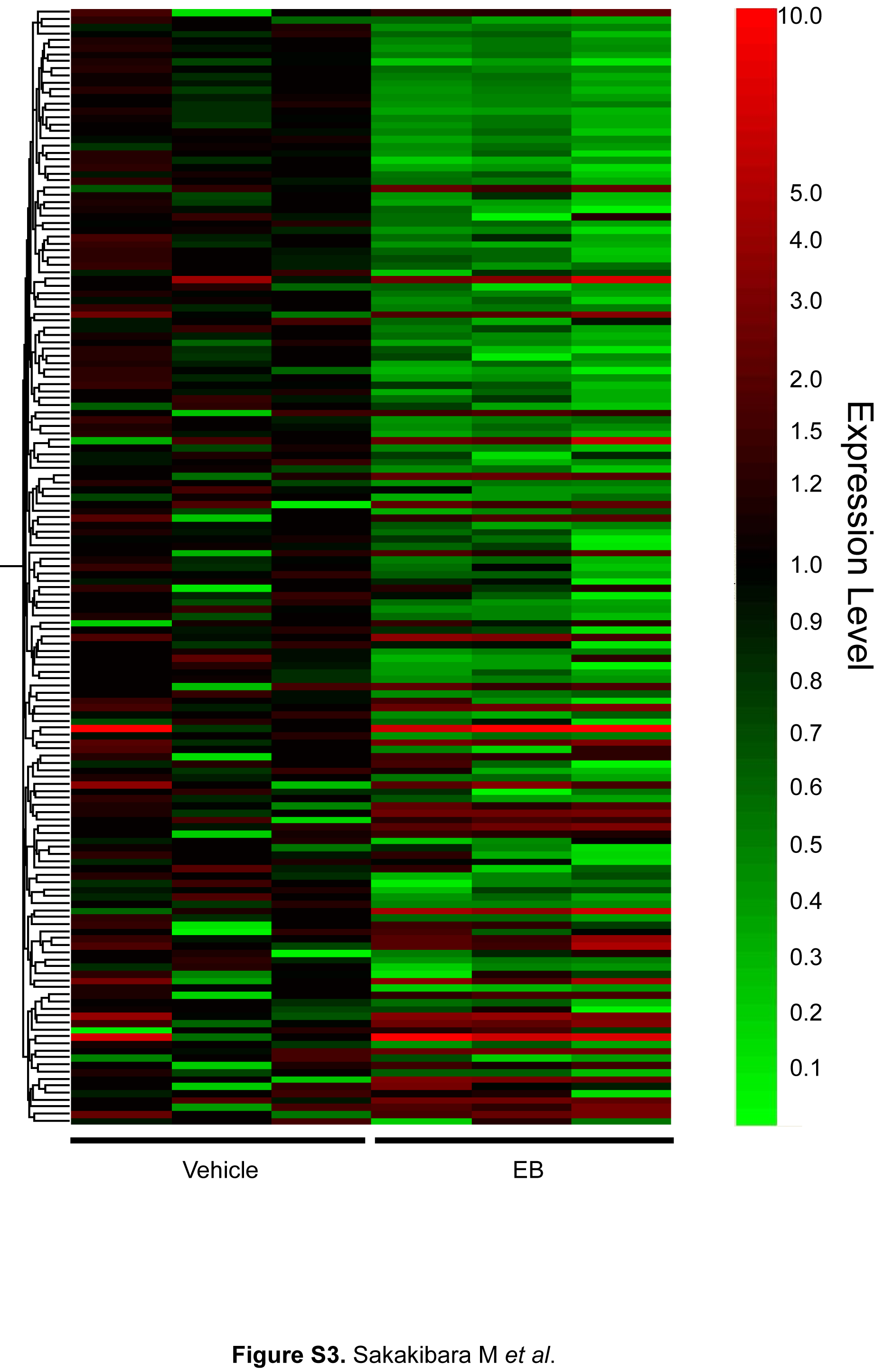

Supplement: Figure S3 — Transcriptional response to 3-h EB treatment in the hypothalamus of neonatal female mice. Heat map shows the hierarchical clustering of 159 Affymetrix probe ID either increased or suppressed by 2-fold or more in the whole hypothalamus of neonatal female mice 3 h after subcutaneous EB injection compared with vehicle injection. See Figure S2 for details. (TIF) [file pone.0079437.s003.tif]

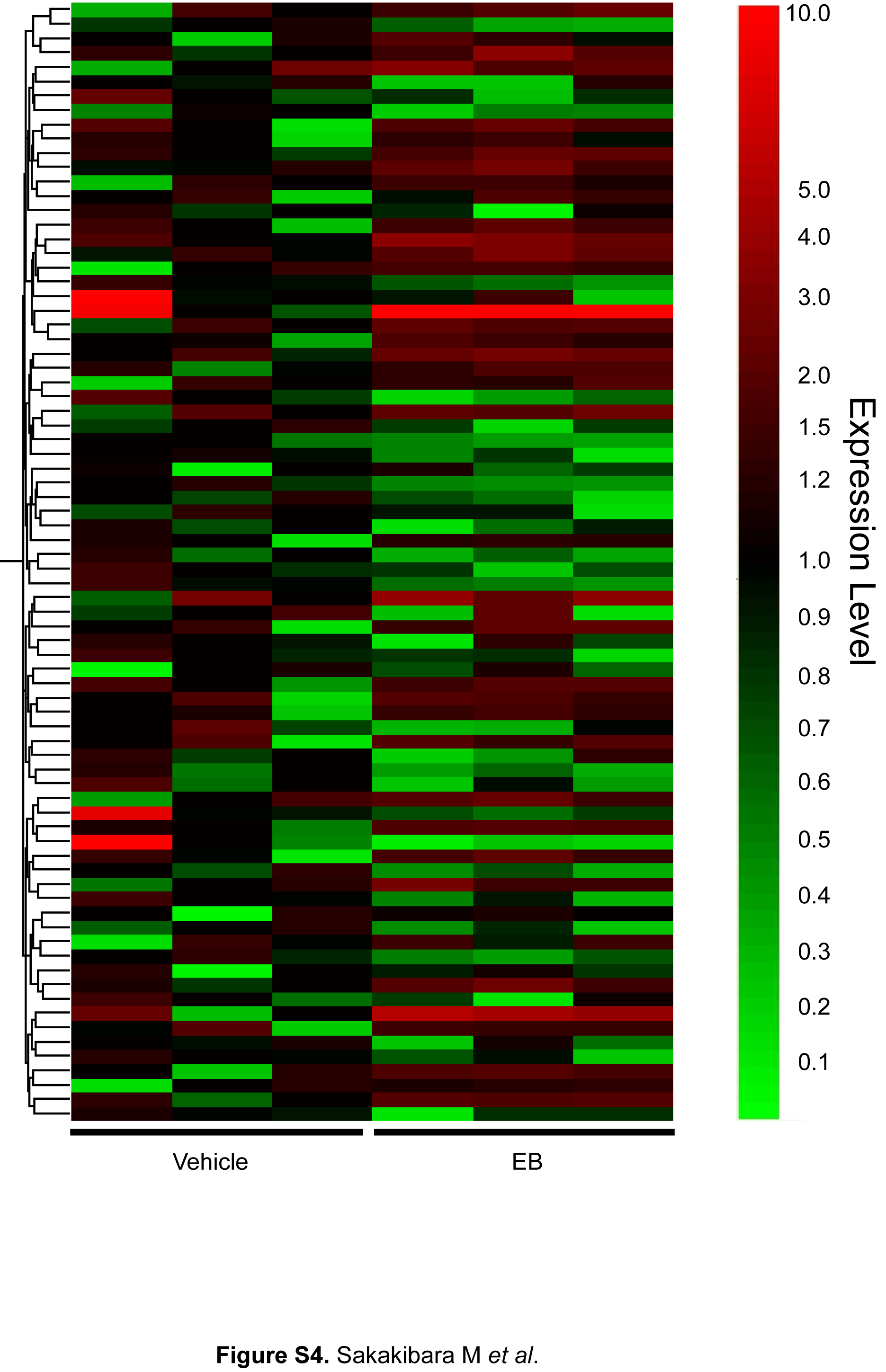

Supplement: Figure S4 — Transcriptional response to 6-h EB treatment in the hypothalamus of neonatal female mice. Heat map shows the hierarchical clustering of 78 Affymetrix probe ID either increased or suppressed by 2-fold or more in the whole hypothalamus of neonatal female mice 6 h after subcutaneous EB injection compared with vehicle injection. See Figure S2 for details. (TIF) [file pone.0079437.s004.tif]

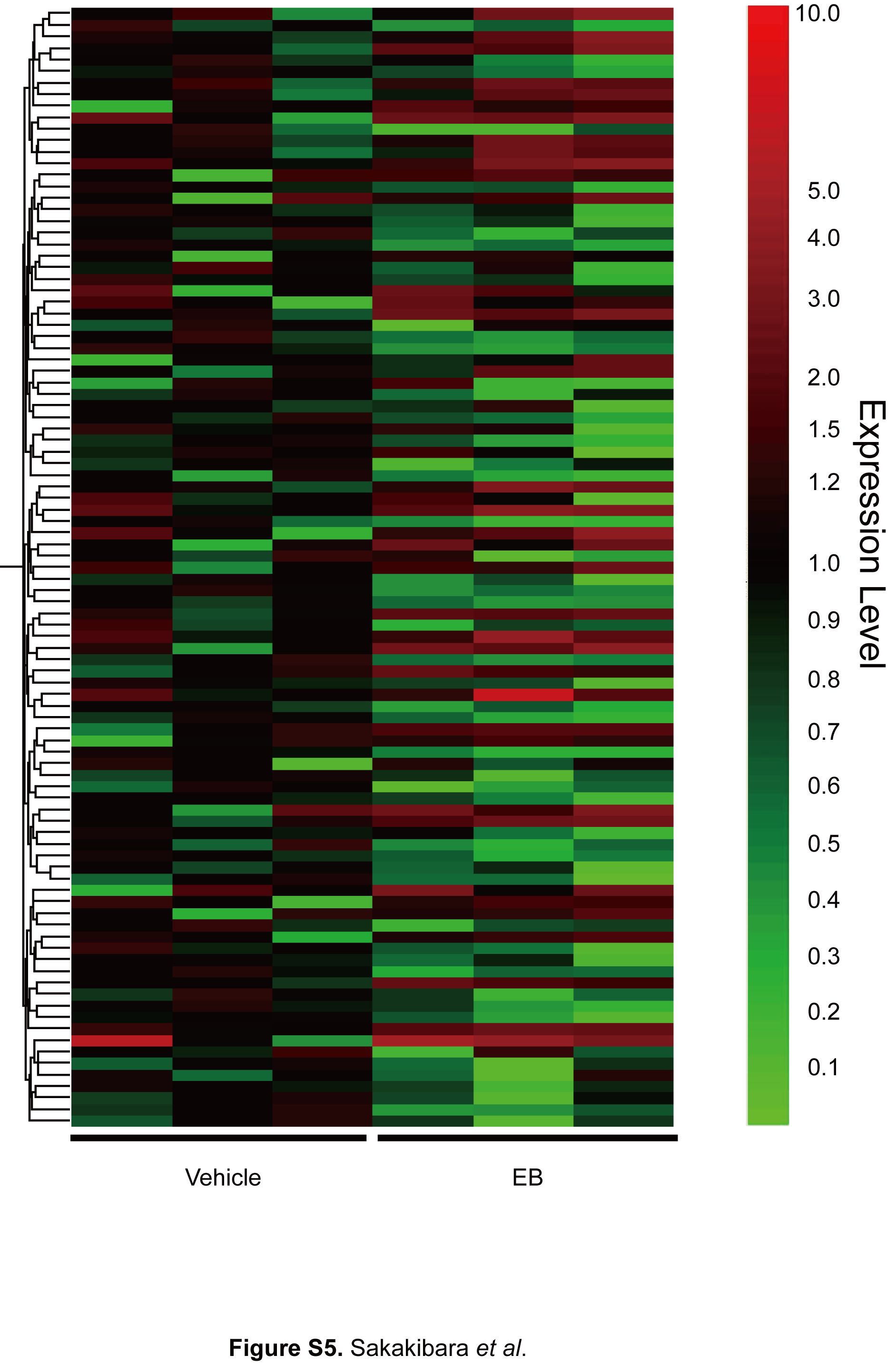

Supplement: Figure S5 — Transcriptional response to 12-h EB treatment in the hypothalamus of neonatal female mice. Heat map shows the hierarchical clustering of 97 Affymetrix probe ID either increased or suppressed by 2-fold or more in the whole hypothalamus of neonatal female mice 12 h after subcutaneous EB injection compared with vehicle injection. See Figure S2 for details. (TIF) [file pone.0079437.s005.tif]

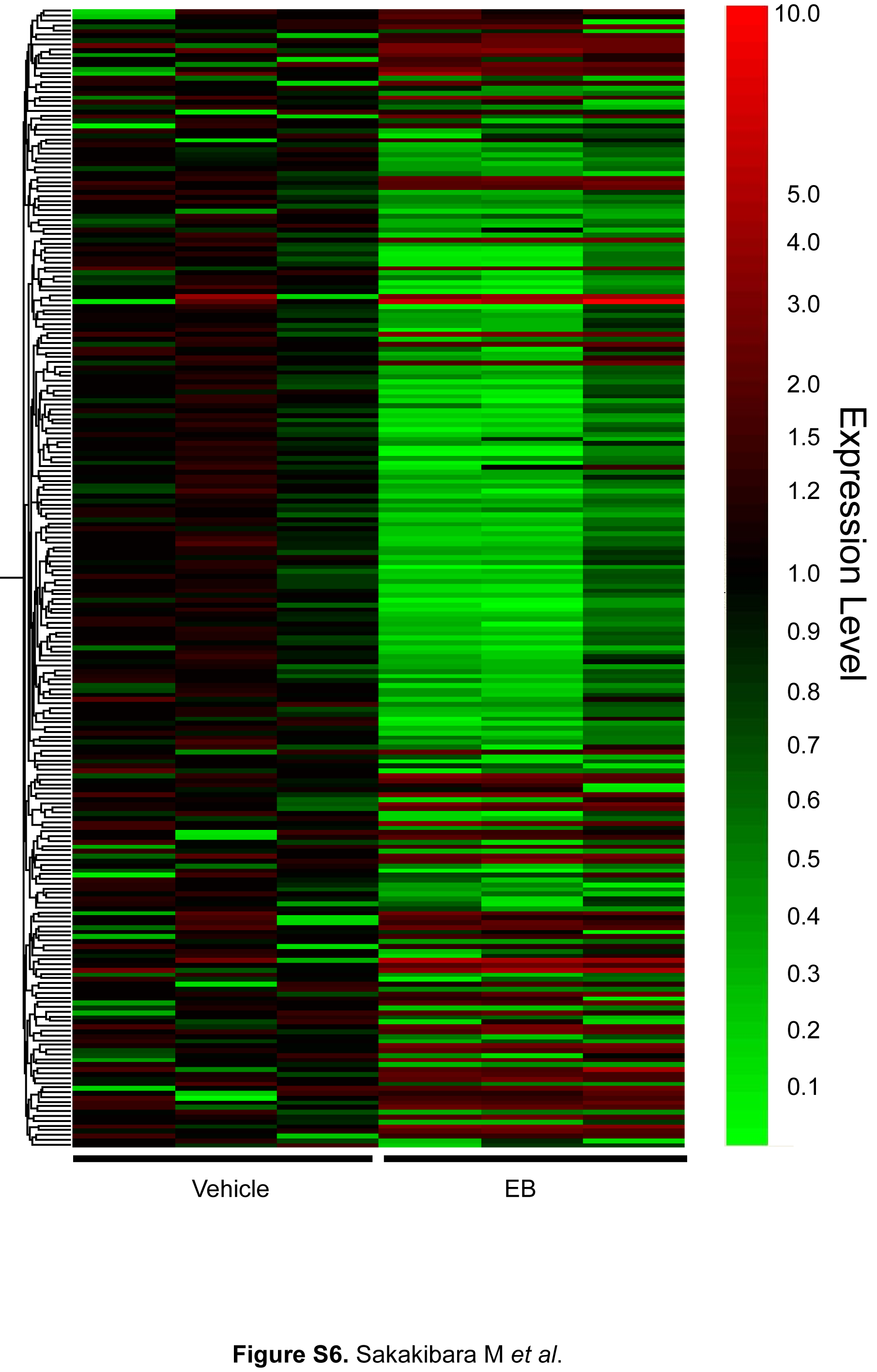

Supplement: Figure S6 — Transcriptional response to 24-h EB treatment in the hypothalamus of neonatal female mice. Heat map shows the hierarchical clustering of 240 Affymetrix probe ID either increased or suppressed by 2-fold or more in the whole hypothalamus of neonatal female mice 24 h after subcutaneous EB injection compared with vehicle injection. See Figure S2 for details. (TIF) [file pone.0079437.s006.tif]
