# Supplementary material for: Microarray Analysis of Perinatal-Estrogen-Induced Changes in Gene Expression Related to Brain Sexual Differentiation in Mice
Source: PLoS One. 2013 Nov 4;8(11):e79437. doi: 10.1371/journal.pone.0079437 (PMC3817063; doi:10.1371/journal.pone.0079437)
Supplement: Table S1 — Sequence of primers used for RT-PCR. (DOC) [file pone.0079437.s007.doc]

**Table S1**. **Sequence of primers used for RT-PCR**

| Genes | Oligonucleotide primer sequences (5’→3’) |  | Product size (bp) |
| --- | --- | --- | --- |
|  | Forward | Reverse |  |
| *Tmed2* | TACACATTTGCAGCCCACAT | TCCCGGACTTCCATGTACTC | 226 |
| *Klc1* | CCTGGTGGAAGAGAAATCCA | CTGTACACCAGGGCCAAGAT | 608 |
| *Nedd4* | CCCTGATAAGCTGCCAAGAG | AGCATGGTGGTGGCTAAATC | 299 |
| *Neurod6* | GGTCCCAAGAGAGGATCACA | ATTCGGGCATTACGACAGAC | 288 |
| *Hcrt* | GGGTATTTGGACCACTGCAC | AGTTCGTAGAGACGGCAGGA | 218 |
| *Pitx2* | CCTCACCCTTCTGTCACCAT | CACCATGCTGGACGACATAC | 634 |
| *Meis2* | CACCCGTTGTTTCCTCTGTT | TTGTGGAGGAGCCTGAAAGT | 378 |
| *Apod* | CCACAGCCAAAGGACAAAAT | TGGCTTCACCCTTTACTTGG | 236 |
| *Vtn* | AGTGGAGCAACAGGAGGAGA | CTCAATGCCCCAGACATCTT | 219 |
| *Ptgds* | AGTGGTGGAGGCCAACTATG | CCAGCCCTCTGACTGACTTC | 245 |
| *Fn1* | AATGGAAAAGGGGAATGGAC | CTCGGTTGTCCTTCTTGCTC | 243 |
| *Igf2* | GTCGATGTTGGTGCTTCTCA | AAGCAGCACTCTTCCACGAT | 194 |
| *Esr1* | ACCGCCCATGATCTATTCTG | TTCCTGTCCAGGAGCAAGTT | 270 |
| *Pgr* | CCTTACCATGTGGCAAATCC | AAATTCCACAGCCAGTGTCC | 322 |
